# Employing a Phased, Interdisciplinary Approach Across Healthcare and School Settings: mHealth Adaptations for Youth with Autism Spectrum Disorder At-Risk of Experiencing Obesity

**DOI:** 10.1007/s10803-024-06666-y

**Published:** 2025-01-04

**Authors:** Caroline Emerson, Caitlin Koob, Kerry Sease, Sarah Griffin

**Affiliations:** 1 College of Health Professions, Medical University of South Carolina, Charleston, SC USA; 2 Bradshaw Institute for Community Child Health and Advocacy, Prisma Health, Greenville, SC USA; 3 Institute for Advancement of Community Health, Furman University, Greenville, SC USA; 4 Department of Public Health Sciences, Clemson University, Clemson, SC USA

**Keywords:** Mobile health technology, Pediatric obesity, Autism spectrum disorder, Rehabilitation therapies, Family-centered care

## Abstract

Youth with autism spectrum disorder (ASD) are at nearly twice the risk of experiencing obesity, compared to youth without ASD. **W**ellness **E**ducation to **C**reate **H**ealthy habits and **A**ctions to **T**hrive (**WE CHAT**) is a novel chatbot that engages participants to enhance primary care delivery and associated care coordination services through mobile health (mHealth) technology focused on social determinants of health (SDOH) and social-emotional health. This study examines multiple perspectives regarding the development and implementation of innovative mHealth technology among youth with ASD. The phases of this study include (1) discussion among individuals and parents of children with ASD, (2) in-depth interviews with primary care providers (PCPs) who treat youth with ASD, and (3) in-depth interviews with interdisciplinary rehabilitation providers who treat youth with ASD. Phases 1 and 2 employed rapid qualitative analysis, and Phase 3 involved inductive thematic analysis to provide context to gaps identified in prior phases. Key themes across the three phases included the *variability of symptoms among individuals with ASD*, the *differences in perceived value of mHealth technology*, the *importance of family-centered care*, and the *role of interdisciplinary support*. Participants recommended the development of branching logic to increase the flexibility of mHealth technology designed for youth with ASD. This study gathered insight from multiple perspectives to identify opportunities for supporting independent participation in mHealth technology while reducing associated caregiver burden among youth with ASD. These findings may inform refinement and expansion of WE CHAT for patients with varying health needs.

## Introduction

South Carolina (SC) has the third-highest pediatric obesity rate nationwide (Child Obesity Action Network, n.d.), and youth with autism spectrum disorder (ASD) are at nearly twice the risk of experiencing obesity, compared to youth without ASD (Levy et al., 2019). Children who are obese are more likely to experience obesity in adulthood and are at greater risk of developing comorbidities such as cardiovascular disease, hypertension, and type 2 diabetes (Kelsey et al., 2014; Simmonds et al., 2015). Since the prevalence of obesity and chronic disease is higher among adults who have ASD, youth with ASD are a particularly important target group for obesity interventions designed to prevent adverse lasting outcomes (Bishop-Fitzpatrick & Rubenstein, 2019; Fowler et al., 2021; Tate et al., 2013). Several studies have demonstrated the success of mobile health (mHealth) obesity interventions in the general population, but little is known about the experiences of youth with ASD with such interventions (Schoeppe et al., 2016; Vandelanotte et al., 2016).

Despite their potential benefits, research indicates that youth with ASD often face challenges when participating mHealth interventions, as partially explained by a variety of coexisting conditions (Askari et al., 2015; Vandelanotte et al., 2016). Reading and comprehension skills, motor development, social attitudes, sensory sensitivities, and behavioral challenges all affect a child with ASD’s participation in mHealth technology (Askari et al., 2015; Baixauli et al., 2021; Bonis & Sawin, 2016; McIntyre et al., 2017; Memari et al., 2015; Oliveira et al., 2021; Pusponegoro et al., 2016). These barriers to participating in mHealth technology also impact their participation in physical activity, contributing to their increased risk of obesity (Fowler et al., 2021; Tate et al., 2013). Further, previous studies have struggled to quantify and contextualize mHealth technology use among youth with ASD due to difficulties with longitudinal data collection and unanticipated challenges, such as perceived pressure on participants and lack of sustained participant interest, that have led to missing data and partial results (Putnam & Mobasher, 2020). However, these challenges are not cause to exclude youth with ASD from ongoing mHealth technology utilization and acceptability research. In fact, research to advance digital health equity suggests that the rapid digitization of healthcare may exacerbate health disparities, without the inclusion of unique populations in this work (Richardson et al., 2022).

Further, as it relates to youth with ASD, family support is critical to participation in mHealth technology uptake and sustained utilization, yet these families often have several compounding challenges that often makes participation in such activities more of a burden than a perceived healthcare tool (Askari et al., 2015; Ayvazoglu et al., 2015; Bonis & Sawin, 2016). Still, existing findings suggest that technology-based programs that focus on the strengths and interests of youth with ASD can improve self-esteem, social engagement, and the ASD community has expressed enthusiasm for mHealth technology in general (Cardon, 2016; Vlachou & Drigas, 2017). Therefore, it is critical to consider the unique needs of youth with ASD—including but not limited to reading level, complex demand of motor skills, and social aspects—should be accounted for from ideation through implementation of mHealth technology interventions (Richardson et al., 2022).

This study intends to address a significant gap in the literature related to the interface between youth with ASD and mHealth technology by incorporating diverse perspectives from home, school, and healthcare environments. The aims of this study are to (1) identify unique attributes of mHealth technology designed to be accessible to youth with ASD, and (2) identify adaptations for existing mHealth technology to improve inclusivity, accessibility, and engagement among youth with ASD.

## Methods

### Intervention and Theoretical Implementation Among Youth with ASD

WE CHAT, which stands for **W**ellness **E**ducation to **C**reate **H**ealth habits and **A**ctions to **T**hrive, is a novel chatbot and healthcare tool that promotes the adoption of healthy lifestyle behaviors among children who are at-risk of experiencing obesity, through an integrated primary care model (Koob et al., 2023, 2024; Koob, Stuenkel, Richardson, Fair, Griffin et al., 2022; Koob, Stuenkel, Richardson, Fair, Sease et al., 2022). A “chatbot” is an app where participants engage in direct messaging and the chatbot responds in a conversational manner (Goyal et al., 2018). The American Academy of Pediatrics’ clinical guidelines for pediatric weight management and obesity treatment suggest children with obesity should attend weekly follow-up visits with their PCP, which is often difficult for the provider or the child and their family (Hampl et al., 2023). WE CHAT is designed to supplement primary care services, providing weekly messaging to support progress towards patient-specific SMART (Specific, Measurable, Actionable, Realistic, Time-bound) goals in the areas of physical activity, nutrition, sleep hygiene, and screen time (Deslippe et al., 2023; Martin et al., 2019). Additionally, WE CHAT offers patients opportunities to escalate their level of care and flag their PCPs for needs related to social determinants of health (SDOH) and social-emotional health. To optimize accessibility, this referral-based chatbot is free of cost and is available via text messaging or email. Each week on the patient’s preferred day, participants receive a link to their weekly WE CHAT session via text or email, as indicated upon enrollment. The link to their weekly WE CHAT session directly opens a browser where the child and/or their guardian provides a combination of multiple choice and open-ended responses to the chatbot’s prompts. For instance, if the child previously set a SMART goal in the area of nutrition, the chatbot will ask about their progress with this goal, the perceived level of difficulty in attaining this goal (and provide an opportunity to choose a more appropriate goal as needed), assess SDOH and/or social-emotional needs and flag the child’s PCP as needed, and provide nutrition-related resources, such as healthy recipes or family-friendly websites (Koob, Stuenkel, Richardson, Fair, Sease et al., 2022). SDOH and social emotional screeners are built into the chatbot algorithm and are assessed approximately once per month, as the patient progresses with their self-selected goal areas (i.e. nutrition, physical activity, sleep, screen time) throughout the duration of the program. Based on rate of patient progress, the WE CHAT program takes approximately six to twelve months to complete.

At this time, patients who are aged 2 to 18 years and who are *≥* 85th weight percentile are eligible for referral to WE CHAT. WE CHAT has been implemented systemwide among children experiencing obesity since May 2022 (Koob et al., 2023, 2024; Koob, Stuenkel, Richardson, Fair, Griffin et al., 2022; Koob, Stuenkel, Richardson, Fair, Sease et al., 2022). This study builds from previous findings to prepare for its implementation among youth with ASD.

### Participants and Data Collection

This three-phased, qualitative study was designed to better understand the needs of youth with ASD and their experiences with mHealth technology from multiple perspectives. This virtual, qualitative approach allowed participants to elaborate on their experiences and facilitated statewide participation (Table 1) (Archibald et al., 2019).

**Table 1:** Participants by phase of study

Phase	1	2	3
*N*	6	4	12
Participants’ Description	Parents of children and youth with ASD, Individuals with ASD	Physicians who treat children and youth with ASD	PTs, OTs, and SLPs that work with children with ASD in a school or healthcare setting
Expertise and Significance to the Study	The lived experience that these individuals shared was critical to the findings of this study, and informed in-depth interviews in subsequent phases. All were familiar with mHealth technology and had experience using it themselves and/or with their child.	All participants were familiar with mHealth technology and routinely treat children with ASD. Two of the four participants were familiar with and had referred patients to WE CHAT. These physicians all held leadership positions within their practice, and 75% held leadership positions in state-level organizations.	All participants were familiar with mHealth technology and routinely treat children with ASD. Therapists were not familiar with WE CHAT but were provided with a brief description of the mHealth technology, and discussed opportunities for its integration into their clinical practice.

Phase 1 involved a meeting with individuals who have ASD (> 18 years old) and caregivers of youth with ASD. A local Patient Engagement Studio (PES) embedded within a large local healthcare system and medical school helped to recruit participants (*N* = 6). Phase 1 involved a community-based, focus group style meeting was conducted through PES within this health system, via Webex. This phase was considered exempt from IRB approval due to its exploratory design to internally generate innovative ideas within the health system; however, insights from this meeting were critical to the foundation of this research. All participants provided verbal consent prior to participation and received $50 gift cards as incentives. This research team presented the concept of WE CHAT and implementation of mHealth technology among children and youth with ASD, and then opened the floor for conversation. Guided discussion questions were provided, and topics included prior mHealth technology experiences, ASD symptomology (i.e. reading and cognition skills, motor skills, social interaction skills), transportation, and nutrition. The questions were co-moderated by the program manager of PES and two researchers (CE, CK), and in total, the discussion lasted approximately 60 min. The discussion was not recorded, but a staff member of PES transcribed the conversation as able and provided a report of the summarized findings and associated quotations for analysis.

Phase 2 involved semi-structured interviews with pediatric PCPs within a large healthcare system in SC. A provider (KS) within this health system led WE CHAT development and implementation and identified PCPs who treat youth with ASD and are familiar with mHealth interventions. Interviewees were contacted by email and invited to participate in an interview, via snowball sampling (*N* = 4). Implementing insight from PES participants, an interview guide was developed for PCPs that focused on prior experiences with mHealth technology, referral considerations, and factors related to ASD that impact children’s experience with mHealth technology (Table 2). Phase 2 interviews were conducted via Microsoft Teams between November 4, 2022 and November 11, 2022. Participants provided verbal consent at initiation of the interview. Interviews were audio-recorded and transcribed by an external transcription service. The duration averaged approximately 20 min per interview.

**Table 2:** Primary healthcare provider interview guide

Primary interview question	Additional probes and prompts
What mobile health technology applications (if any) have you suggested for patients to try?	• Apps like “Habitz: Kids Learn Good Habits,” “Exercise: At Home Workout App,” and “Exercise for Kids at Home” are some current examples of mobile health technology applications designed for children (Lee et al., 2023). • How do you identify which patients you would suggest these applications for? • Thinking about reading level and cognition, how do you decide which children would benefit from independently using a mobile health app? • Is there a minimum age for participation that you have in mind? • Would co-engagement from a parent allow even younger children to participate? • What might prevent you from recommending a mobile health app to a patient? • Have patients ever expressed concerns about the programs you suggest? • Is there a way for families who don’t have internet access at home to participate? OR • What has kept you from recommending the use of mobile health technology? • What are some alternative health interventions that you would recommend?
Have you suggested a mobile health app to families of children with autism?	• Was the app designed to be used by the child, the parent, or both? • What are the factors you consider when you are suggesting a mobile health app to families of children with autism? • How might a child with autism’s motor skills impact their experience with mobile health technology? • What are some examples of physical activities that could be suggested for people who have lower motor skills? • How might a child’s social interaction skills impact their experience with mobile health technology? • What are some good individual physical activities for when group play is not possible? • What are some ways to foster social interaction skills when using mobile health technology? • Apps like “My Food – Nutrition for Kids” help children learn about good nutrition. How do these apps help or benefit children who are picky eaters due to food sensory problems? • What are some methods that are useful for reducing food sensory issues? • What concerns do you have about interventions that involve specific nutrition plans, particularly related to if they pose a risk for interrupting instinctual hunger cues? • What concerns do you have about children developing or strengthening cravings or overindulging when given the opportunity? • Are there any other barriers you would anticipate for a child with autism concerning the structure or content of existing mobile health technology? • Would co-engagement in the program with a parent address these concerns?
How might limited access to transportation impact a child’s participation with mobile health technology?	• How could we help families overcome the barrier of transportation?
How might access to the built environment (parks, playgrounds, sidewalks, etc.) in a neighborhood impact participation with mobile health technology?	• What alternative physical activities would you suggest to a family who does not have access to these play spaces?

Lastly, informed by preliminary review of previous findings, Phase 3 focused on perspectives of rehabilitation therapists and their potential to influence WE CHAT adoption and sustainability among youth with ASD (*N* = 12). Physical therapists (PT), occupational therapists (OT), and speech language pathologists (SLP) from one region of SC were interviewed to share their experiences with mHealth technology in treating patients with ASD. Recruitment was initiated by contacting OTs with professional contacts with this research team and continued via snowball sampling until saturation was met. Participants were required to work in either a healthcare or school-based setting as an OT, PT, or SLP in SC. There were no further exclusion criteria for this study. The study included therapists from school (*n* = 4) and healthcare (*n* = 8) settings. Of note, some SLPs received specialized training in avoidant and restrictive food intake disorder, which is not uncommon among children with ASD and may complicate obesity interventions for children with ASD, providing a critical lens to addressing nutrition goals among this population (Willmott et al., 2024).

The Phase 3 interview guide, informed by preliminary findings of Phases 1 and 2, was modified to include the use of mHealth technology in therapy sessions, the role of mHealth technology in home exercise programs (HEPs), and suggested adaptations for existing mHealth technology for youth with ASD. The interview also examined the roles of collaborative care and family systems in the support of youth with ASD (Table 3). The duration averaged approximately 30 min per interview. All interviews were audio-recorded via Microsoft Teams and transcribed by a professional transcription service. Interviewees in Phases 2 and 3 volunteered their time and did not receive incentives for participation; however, all participants provided appropriate consent prior to participation. All phases were approved by the [Institution Blinded for Review] Institutional Review Board.

**Table 3:** Rehabilitation therapy provider interview guide

Primary interview question	Additional probes and prompts
Generally, how have you used health technology in your therapy treatment?	• Tell me more about that. OR • If you have not, what keeps you from using health technology in your therapy treatment? Tell me more about that.
What mobile health technology (if any) have you incorporated into therapy sessions?	• Probe: Apps like “Habitz: Kids Learn Good Habits,” “Exercise: At Home Workout App,” and “Exercise for Kids at home” are some current examples of mobile health technology applications designed for children. • How have your patients’ benefited from incorporating mobile health technology into your treatment? • How do you identify which patients might benefit from treatment involving mobile health technology? OR • What has kept you from using mobile health technology during therapy sessions? • What are your preferred resources to use during a therapy session? How do you believe these resources benefit patients, compared to mobile health technology?
Do you provide home exercise programs for your patients to perform outside of therapy? (Note: With or without technology) Why or why not?	• How (if ever) have you incorporated mobile health technology as part of these home exercise programs? • Who is typically responsible for carrying out home exercise programs between therapy sessions? (Probes: Caregiver/parent, patient, etc.) • What might support parents when trying to carry over a patient’s home exercise program? • What reservations, if any, do you have about offering a home exercise program?
How have you worked collaboratively with other healthcare professionals to design a patient care plan?	• Probe: Physicians, physical therapists, occupational therapists, speech language pathologists • Tell me more about that. • Which healthcare professionals do you work most closely with? Why do you think that is? (Probe: overlap in scope of practice, co-treatment, etc.) • Could you provide some examples of times you’ve participated in collaborative care, specifically for children/adolescents with autism? • How do you think therapy outcomes among children/adolescents with autism are influenced by collaborative care?
In previous discussions with individuals with autism, parents of children with autism, and other medical providers, it was frequently mentioned that autism is “highly variable,” and each child with an autism diagnosis presents differently. Children with autism differ in their cognition, fine motor skill ability, social skills, and eating behaviors. However, existing mobile health technology does not always account for these differences. How do you think mobile health technology could be adapted to account for these changes?	• If you were to develop/adapt a mobile health technology for use among children/adolescents with autism, what would your top 3 priorities be? • What are your thoughts on providing multiple options within each prompt so that the child can choose what is appropriate for them? (Example: physical activity prompt with options to jump with or without a jump rope) • How many options would be appropriate? Would more than two be overwhelming? • What are your thoughts on adding parameter questions to customize the child’s experience? Prompts would be filtered to match the selected preferences and ability levels. • What other ideas do you have for customizing mobile health technology for children with autism?
Children with autism may require co-engagement or support from a parent to successfully use mobile health technology. What could you do during a therapy session to help lighten the load on these parents?	• In apps that do not provide the option to customize, what do you think about modifying an app’s recommendations during a therapy session so that the child could participate at home? (Probe(s): grading the activity, incorporating patient interests, etc.) • What app features would facilitate a child’s ability to participate independently? • What other barriers would you anticipate for a child with autism concerning the structure or content of existing mobile health technology?

### Data Analysis

Two qualitative methods were employed to analyze data across phases: (1) Rapid and Rigorous Qualitative Data Analysis, or RADaR, and (2) inductive thematic analysis. Transcriptions from Phases 1 and 2 underwent RADaR, an applied, systematic qualitative analysis strategy often used to evaluate findings within a dynamic healthcare environment (Keniston et al., 2023; Watkins, 2017). This process was done chronologically, analyzing Phase 1 data immediately following data collection, using preliminary findings to inform data collection for Phase 2, and subsequently repeating the process prior to Phase 3.

Phase 3 transcripts were reviewed, coded, and analyzed via inductive thematic analysis, using ATLAS.ti software, by the research team (Braun & Clarke, 2021; Paulus & Lester, 2016; Soratto et al., 2020). Two independent coders developed an initial codebook based on findings from Phases 1 and 2, and several codes were added throughout the coding process as themes emerged. Transcripts were independently coded, with three out of twelve interviews randomly selected and double-coded to establish inter-coder reliability. Coders, who were trained in qualitative research methods and analysis, identified and discussed discrepancies to reach a mutual consensus on the appropriate coding scheme. Major themes, as identified via frequency of codes, was used to finalize key themes and identify similarities and differences across settings. Illustrative quotes across the three phases were selected and presented to support thematic findings.

## Results

Key themes, woven across the three phases, included the *variability of symptoms among individuals with ASD*, the *differences in perceived value of mHealth technology*, the *importance of family-centered care*, and the *role of interdisciplinary support* (Table 4).

**Table 4:** Summary of findings by theme

Theme	Subthemes	Additional quotes
Autism Variability	• Range of reading and cognition levels • Range of motor skill levels • **Relationship between social skills and safety** • Differences in desired auditory or visual sensory experience • **Recommendations that account for the variability of ASD**	• It is important “that directions are very simple and explicit. I would, at the beginning of any new activity…have built in a couple of models with descriptions or a narrative about, you know, what they’re doing and what the child’s going to do” -*School SLP* • “And then maybe, you know, once a week or every two weeks, you kinda reset the parameters based on how they’re progressing.” -*Healthcare PT*
Variability in Perceived Value of mHealth Technology	• **Range of benefits (increased independence**,** schedule integration**,** attention capturing**,** and improved self-awareness)** • **Useful tool for teletherapy and home exercise plans** • Increased familiarity following the COVID-19 Pandemic • Too expensive • **Limited time to use mHealth technology during sessions** • **Considered a last resort when preferred methods are not effective**	• “HEP2GO is a big part of that, and they have—it’s a, kind of a database of common home exercises or things that are worked on in OT or PT sessions…It reduces the load on the therapist to be able to make handouts.” *-Healthcare OT* • “So, again, personally, I don’t think I would use it as a PT during my session unless I had a child who was difficult to engage with.” -*Healthcare PT* • “I have very limited time with my students. And I feel like, across the world, people are using the computer and the screen and the iPad and the tablets and the games way more than we all need to be.” -*School OT*
Family-Centered Care	• **Importance of parent support with home exercise programs** • Caregivers may struggle to offer support due to financial strain, psychological strain, and lack of time. • **Suggestions for reducing caregiver load** (simple tasks, already completed during therapy, clear instructions, explaining the purpose, recommendation programs that match the family’s lifestyle, visual demonstrations and reward system to limit child’s reliance on caregivers)	• “I always give some sort of home exercise program…But if there’s not carryover at home, then—which, sometimes, there’s not carryover at home, and you know, you see kids be more successful if there is.” -*Healthcare PT* • “I put a lot of work into some of my home exercise programs with totally no response from the parents.” *-School OT* • “The families are busy…I’ve realized that like I just sometimes need to stick with just one thing…just make it something that is really simple, really concrete, something that they could easily incorporate into their daily routines.” *-Healthcare SLP* • “Clear directions and impact into what functional skill you’re actually addressing. Because sometimes, home exercise programs don’t make sense to caregivers about why would you—you know, why would you recommend that a patient do yoga every day this week, and what is that functionally gonna impact?” -*Healthcare OT* • “Linking whatever is on that app to common areas of special interest, because that’s gonna harness motivation” -*Healthcare OT* • “And actually demonstrate with narrative what the child should do, so, they have that visual and can see what they’re supposed to be doing before they begin themselves.” *-School SLP* • “Stars or badges for activities, which go on their profile and people could cheer them on.” -*Patient Expert*
Interdisciplinary Support	• **Providing efficient care in clinical settings** • **Collaborative plans that reduce caregiver burden** • Barriers to collaboration (HIPAA and insurance) • Less frequent collaboration in school settings	• “A lot of our patients overlap since we have speech, occupational therapy, and physical therapy in the clinic. So, there are several patients that we kind of—the speech therapist, PT, and OT will all kinda talk about patterns that we’re noticing or things that kind of overlap.” -*Healthcare PT* • “With the teachers, like general ed or special education, if I’m just really excited about how they’re doing or successes that I’ve found in therapy, I’ll show them what we did, how we did it, how we—you know, the modifications we did.” -*School OT* • “If we are working collaboratively on the same patient, having clear discussion about roles and goals for kids is key so that you can create overlapping home exercise programs…if, for example, the child gets OT, PT, and speech, having three distinct home exercise programs is too much for one family.” *-Healthcare OT*

Note: Bolded subthemes are frequently repeated and described in detail in the Results

### Theme 1: Variability of ASD Symptomology

The variability of ASD symptomology was described in several ways, including differences in reading and cognition levels, motor skills, social skills, and desired sensory experiences. The relationship between social skills and safety was most frequently mentioned in relation to a child’s engagement with mHealth technology. PCPs expressed that youth with ASD interpret and follow instructions in a literal sense and may have to be taught concepts that are inferred by their peers without ASD. One patient expert with ASD reflected on his own struggles related to social skills and having the awareness to make appropriate safety decisions. In response, parents of children with ASD emphasized the need for clear instructions related to safety within mHealth technology for youth with ASD.

“‘Check with your parent first’ should be a prompt before instructing a child or adolescent to, for example, go on a bike ride.” -*Patient Expert*.

Therapists considered how literal interpretation could impact the child’s ability to complete exercises correctly. Therapists emphasized the importance of clear, detailed instructions and the use of visuals as a tool to improve understanding.

Further, beliefs related to the variability of ASD were reflected in recommendations for developing a mHealth app for youth with ASD. Patient experts proposed “this or that” options in which the individual could choose the activity that best fits their ability level. Therapists agreed with providing the element of choice but contemplated the appropriate number of choices.

“My nephew, who is autistic, you could give him 15 choices and he would find the one he wants…but then I have other kids that – I mean even making a choice is so anxiety-producing that it would be hard.” -*Healthcare OT*.

Patient experts also suggested optional “parameter identifier questions” to account for the variability of ASD symptomology. Per patient experts, ideally, mHealth technology users would have a personalized experience by only receiving prompts relevant to their selected parameters. Therapists emphasized the importance of having the caregiver or therapist adjust parameters as the child progresses. There is no “one-size-fits-all” app for youth with ASD, per PCP’s perspectives. Due to the variability of symptoms, flexibility would be crucial for any mHealth technology designed for youth with ASD.

### Theme 2: Variability in Perceived Value of mHealth Technology

Participants in each phase varied in their experience levels and perceived value of mHealth technology. Some patient experts considered mHealth technologies useful for “facilitating independence” and assisting with transition times through schedule integration. Patient experts reported using wearable mHealth technologies, identifying Fitbits, apple watches, TracknShare, and mindfulness apps, such as Headspace or Calm, as tools for emotional regulation among youth with ASD. Some PCPs valued mHealth technology, as one PCP lead the development of WE CHAT, and another PCP described her use of apps for behavior intervention and websites to assist children with ASD with food sensitivities. Several therapists also considered mHealth technology a useful tool for capturing and maintaining the attention of youth with ASD, motivating participation, and providing visual feedback to improve self-awareness.

With the Articulation Station app, you can pick whichever sound you’re targeting with that patient…And I particularly like it for my patients because you get to record yourself as you’re practicing the word, and so it helps them with just a like motivation; they always want to record themselves, but also too like self-monitoring skills and being able to hear how they said it, identify if they said it correctly, and then like increase their self-monitoring and self-awareness skills.

-*Healthcare SLP*.

Many therapists recognized the value of mHealth technology in relation to teletherapy and HEPs. Virtual options for HEPs were perceived as affordable, accessible, and convenient for children with ASD and their families.

There are a few local yoga classes in the area I have recommended, but there’s limitations in that as, obviously, cost, time…So, I tend to recommend the apps and the electronic options as, like, to probably 75% of my families, I would say.

-*Healthcare OT*.

In contrast, some patient experts and PCPs felt that mHealth technology was not worth the financial cost or the additional strain on parents for setup and logistical use.

With respect to many types of technology aimed towards people with ASD, remembering to bring charged watches, phones, and tablets adds, one more layer of things that we need to do.

-*Patient Expert*.

“I don’t think you should have to pay to be healthy. It should be free.” -*PCP*.

Although PCPs suggested youth with ASD use mHealth technology collaboratively with their therapist, some therapists preferred traditional therapy methods and only valued mHealth technology as a last resort if their preferred methods were not effective. In the school setting, therapists reported that additional support with mHealth technology may not be feasible at times due to typically shorter, less frequent sessions, and their focus on specific school-oriented goals in comparison to clinical sessions.

Still, most therapists were not against the development of mHealth technology for youth with ASD. If affordable and beneficial apps were developed for youth with ASD, therapists expressed their willingness to implement mHealth technology as they would any other therapeutic intervention.

“If there was an app that was successful for them, I would definitely use it during therapy, practice with it just like a lot of speech therapists will use an AAC device in speech therapy to help them.” -*Healthcare PT*.

### Theme 3: Family-Centered Care

Across all phases, participants discussed why parental involvement is key for the success of youth with ASD. Therapists specifically recognized the value of HEPs and discussed how these plans are often unsuccessful without caregiver support.

Participants described a variety of reasons why caregivers may struggle to provide additional support, including barriers related to cost, time, and effort. Therapists acknowledged barriers to participation and suggested several ways to encourage participation while reducing the load on caregivers. One common suggestion was to assign minimal HEPs that could easily be incorporated into the family’s daily routine. Therapists found that communicating clear instructions and explaining the purpose of HEPs resulted in better carryover to the home environment, promoting children’s independence in functional activities across settings. Another common suggestion was to only assign tasks that have been successfully completed during a therapy session.

I try and make it something too that if it was easier or easy for the child to do in therapy with me, that I’m going to send it home for home programming. If it was hard for me to get success in that activity during my own treatment, using the skills and knowledge that I have, that I’m not going to give it for home programming to the family.

-Healthcare SLP.

Providers reported that a family’s familiarity with technology influenced their recommendations. One PCP pointed out that when families ask about technology, that provides him with a cue that a mHealth technology could fit their lifestyle. This suggestion reiterates therapists’ recognition of providing HEPs that fit a family’s lifestyle rather than introducing stress-inducing activities.

“It’s a combination between evaluating the fit between the client’s own intrinsic capacities…and also—I mean, whether or not that technology will adapt into their life well.” -*Healthcare OT*.

Therapists suggested several mHealth app adaptations that could facilitate independence for youth with ASD. Per therapists, there may be less strain on the caregiver to encourage participation if the child is engaged by visual models or activities related to patient-specific interests. Therapists also suggested reward systems may keep youth with ASD motivated to participate consistently, while decreasing their reliance on caregivers for step-by-step support.

Many children who have ASD are also in ABA therapy and a lot of those work off of a behavioral model. And so, having some sort of internal reward chart or level up experience may also be motivating to move it from an HEP into more of a game-like experience.

-*Healthcare OT*.

### Theme 4: Interdisciplinary Support

PCPs advocated for interdisciplinary collaboration to best support youth with ASD. PTs, OTs, and SLPs were all mentioned as vital resources. When reflecting on their experiences with interdisciplinary care, therapists working in the clinical setting reported working with other PTs, OTs, and SLPs within their clinic.

In school settings, therapists reported they may work for multiple schools and have limited interaction with other therapists. Interdisciplinary collaboration was more commonly mentioned between teachers and therapists in the school setting, compared to the healthcare setting.

“I interact with speech therapists and physical therapists. Not on a daily basis. The physical therapist I only see maybe, you know, 30 minutes one time a week, because they go to so many different schools than I do.” *-School OT*.

Perceived outcomes related to interdisciplinary care included efficient care and reduced caregiver burden. Therapists shared how youth with ASD can achieve their goals more quickly when interdisciplinary therapists work on different aspects of the same goal. Therapists may also create a collaborative care plan to reduce the load on parents.

“This kid needs to put their shoes on, and the OT’s gonna work on the self-care part of that, the PT’s gonna work on the core strength part, and the speech therapist is gonna work on the direction following part.” -*Healthcare OT*.

## Discussion

Youth with ASD have a significantly higher risk of experiencing obesity than children without ASD (Levy et al., 2019). mHealth technology, such as WE CHAT, may be an effective intervention to prevent and treat pediatric obesity (Koob et al., 2023, 2024; Vandelanotte et al., 2016). However, little is known on how to adapt web-based pediatric health promotion interventions for youth with ASD. To inform adaptations and improve the accessibility of WE CHAT as a resource, this study focused on potential interaction among youth with ASD with a chatbot, like WE CHAT, and patient-, provider-, and family-driven adaptations to optimize accessibility and health outcomes among this population.

### Recommendations to Account for ASD Variability

The range of ASD symptomology reported across all three phases corresponds with previous studies which report varying reading and cognition levels, motor skill levels, and visual and auditory preferences among youth with ASD (Chevalier et al., 2022; McIntyre et al., 2017; Oliveira et al., 2021). Previous research found that the sensory sensitivities of individuals with ASD influenced the results of robot-assisted therapy, suggesting such technologies account for these sensitivities through varying, adjustable levels of intensity for user preferences (Chevalier et al., 2022). WE CHAT developers may consider this recommendation by allowing users to adjust a variety of components to match their preferences, including brightness, color palette, audio-feedback, and sound effects, for a more intuitive, accessible interface.

Further, patient experts recommended the development of branching logic for parameter-setting to allow for this type of flexibility. Per patient experts, users could set their reading level, motor skill level, opt-in or out of safety features, and select visual and auditory preferences during the enrollment process. Software that allows for personalization is already a common feature of interventions designed specifically for people with ASD (Goyal et al., 2018; Van Der Spek et al., 2019). If implemented, WE CHAT could offer their default program as a simple option and develop branching logic for users with ASD who wish to customize their experience, or who may require additional adjustments to participate.

Allowing users to make choices is another element that can make a design more game-like for people with ASD (Whyte et al., 2015). Providing multiple choices within each physical activity or nutrition prompt could make WE CHAT more relevant and motivating for users who have ASD. Based on feedback from therapists, the number of choices per prompt could also be an adjustable parameter.

According to patient experts and PCPs, youth with ASD may interpret directions in a more literal sense than children without ASD. These findings are supported by a study in which participants with ASD struggled to interpret figurative language in comparison to those without ASD (Vulchanova et al., 2019). To ensure users’ safety, WE CHAT developers may consider adding prompts with reminders related to safety, such as asking a parent’s permission before leaving the house. Developers could also include detailed instructions and visuals to increase the likelihood of activities being completed as designed.

### Recommendations to Ease Caregiver Load

Participants reported high caregiver load which was consistent with previous research on parents of children with ASD with lower support needs who reported increased responsibilities and difficulties with meeting the needs of every family member (Ayvazoglu et al., 2015). PCPs recommended working with rehabilitation therapists to guide youth with ASD through their use of mHealth technology and ease the strain on parents. For youth with ASD using WE CHAT, a consult with their rehabilitation therapist may help them get the most benefit out of their mHealth technology experience. While school therapists may be limited in their ability to help with tasks outside of specific school objectives, clinical therapists may have more flexibility to help with WE CHAT set up and support—particularly as it relates to their therapeutic goals for motor development and participation in functional activities.

Across all phases, participants agreed that WE CHAT should require minimal effort from parents. Therapists gave a variety of suggestions in relation to HEPs that could be applied to WE CHAT to reduce caregivers’ load. WE CHAT could allow users to add activities to a list of favorites so that youth with ASD always have some options they can complete without assistance from caregivers.

Therapists suggested how to motivate children with ASD to participate without being reliant on caregiver support. In response, WE CHAT developers could provide attention-capturing visuals and demonstrations to maintain children’s attention and motivation to participate in the program, as has been demonstrated in previous studies (Cardon, 2016; Chevalier et al., 2022; Whyte et al., 2015). WE CHAT developers could add a social media-like element so that select family and friends can provide encouragement about the child’s achievements, such as through a message board. Therapists also recommended a game-based reward system which is supported by the generally positive findings from a systematic review on 30 gamified interventions designed for youth with ASD (Van Der Spek et al., 2019; Whyte et al., 2015). WE CHAT developers could create a game-based reward system in which participants unlock new games as activities are completed.

### Recommendations to Healthcare Systems Utilizing WE CHAT

Collaboration between multiple providers is considered one of the best practices for the treatment of youth with ASD (Hyman et al., 2020). During Phase 3, therapists discussed how collaborative care can increase efficiency and improve outcomes for youth with ASD by allowing professionals to work on multiple components of the same goal. Therapists who collaborate can also lighten caregiver load by designing one plan for a family to follow instead of assigning a multitude of HEPs.

Rather than providing the default recommendations to encourage a healthy lifestyle through WE CHAT participation, therapists could work collaboratively with PCPs and other therapy disciplines to promote referrals and sustained participation in this health promotion programming among youth with ASD who receive therapy within this health system. For example, the PCP may recommend WE CHAT, and therapists could collaborate by adding their own HEPs and activities to that child’s WE CHAT account. This collaborative plan could reduce caregiver load by including all HEPs within one app, and providers’ time spent on family education is billable (Volkmar, 2021). This could also increase the intervention’s efficiency since multiple providers would be working towards the same healthy lifestyle goals.

### Limitations

This study has several limitations. One limitation of this study is that the discussion was largely based on the theoretical implementation of an app. There is a need for further research to determine how WE CHAT would impact pediatric therapy sessions if implemented. WE CHAT would need significant development to incorporate the sophisticated recommendations from this study. WE CHAT is currently only available within one healthcare system, so it may be difficult to make the intervention available to youth with ASD outside of the organization. As WE CHAT evolves, developers are encouraging a self-referral option among community members to improve the reach and accessibility of this program. With time, program expansion may improve WE CHAT’s acceptability, appropriateness, and feasibility among multiple pediatric populations.

## Conclusion

This study gained insight on the factors that impact youth with ASD and their experiences with mHealth technology. Participants highlighted the variability of ASD and the need to develop mHealth technology that reduces caregiver burden. Rehabilitation therapists described their experiences with mHealth technology during therapy and gave suggestions for facilitating independent participation of youth with ASD. Results will inform refinement of an innovative mHealth technology and expand its applicability and inclusivity of patients systemwide. WE CHAT developers could make the resource more beneficial for youth with ASD by developing branching logic that allows users to customize their experience.

Future studies should measure patient- and family-level outcomes of WE CHAT users including the impact on obesity and caregiver satisfaction scores. The outcomes of youth with ASD using WE CHAT should be compared to the outcomes of neurotypical WE CHAT users. As WE CHAT developers increase branching logic, researchers should evaluate how the option to customize influences outcomes on youth with ASD. Future studies should also examine how using WE CHAT alongside a PT, OT, or SLP influences the outcomes of youth with ASD.
